# Beyond the borders—coma as a complication of imported Japanese encephalitis: a case report

**DOI:** 10.1186/s13256-026-06206-5

**Published:** 2026-06-12

**Authors:** Gio Gemelga, Vishwajit Tuchscherer, Jessie Wang, Mrinmayee Naik

**Affiliations:** St. Joseph’s Medical Center, 1800 N California St., Stockton, CA 95204 USA

**Keywords:** Acute encephalopathy, Infectious disease, Japanese encephalitis virus, Coma

## Abstract

**Background:**

Japanese encephalitis (JE) is a mosquito-borne flavivirus infection endemic to much of Asia and the Western Pacific. Although most infections are asymptomatic, less than 1% progress to clinical disease, typically presenting as acute encephalitis. The case fatality rate ranges from 20 to 30%, and up to half of survivors develop long-term neurological or psychiatric sequelae. While uncommon among travelers from non-endemic regions, risk increases with prolonged rural exposure or lack of vaccination. Immunocompromised individuals, including those with hematologic malignancies, may experience severe and rapidly progressive diseases. Awareness of this rare but life-threatening infection is essential when evaluating returning travelers with acute encephalopathy.

**Case presentation:**

A 70-year-old Lao male living in the California Central Valley with a history of multiple myeloma, hypertension, and congestive heart failure presented with progressive confusion and weakness after travel to rural Laos. His trip exceeded the recommended duration, and his Japanese encephalitis virus (JEV) vaccination status was unknown. On admission, he was febrile and encephalopathic with new-onset rigidity. Computed tomography showed chronic calcifications consistent with prior neurocysticercosis, while magnetic resonance imaging revealed bilateral frontal and left insular cortical hyperintensities without thalamic involvement. Cerebrospinal fluid analysis conducted 9 days after his cognitive decline showed elevated protein without pleocytosis, and infectious and autoimmune panels were negative. Despite empiric antibiotics and supportive care, his condition deteriorated to coma, requiring intubation. Serologic testing later confirmed recent JEV infection with positive Immunoglobulin G (IgG) and low-titer Immunoglobulin M (IgM). After multidisciplinary discussions, comfort care was initiated, and he died in inpatient hospice.

**Conclusions:**

This case illustrates the diagnostic challenge of acute encephalopathy in immunocompromised travelers. Myeloma-related immune dysfunction, as suggested by his lack of pleocytosis, likely contributed to impaired viral clearance, leading to rapid progression. The absence of typical thalamic lesions and the presence of frontal and insular involvement represent an uncommon neuroimaging pattern in JE. Pre-existing neurocysticercosis may have further worsened neurologic outcomes, as prior studies suggest JEV co-infection can lead to worse prognosis. Our case highlights the importance of having a broad differential in an undifferentiated patient and the value of investigating a patient’s history holistically. Imported JE should be considered in travelers returning from endemic regions, particularly those who are immunocompromised. A detailed travel history, broad infectious workup, and recognition of atypical imaging findings are key to diagnosis. Vaccination and mosquito precautions remain the most effective preventive measures against this often-fatal disease.

## Introduction

Altered mental status is a clinical presentation that physicians are trained to evaluate using a systematic framework. This approach considers brain dysfunction resulting from structural disease, metabolic derangements, hypoxia, or infection. In most cases, clinicians can identify and treat the underlying cause. However, when this framework fails to establish a diagnosis, it prompts diagnostic curiosity and encourages a deeper investigation into the patient's primary disease process.

We report a fatal case of Japanese encephalitis (JE) in a recent traveler to Laos, underscoring the importance of detailed history-taking and a structured diagnostic approach. JE is an inflammatory brain disease caused by Japanese encephalitis virus (JEV), a flavivirus primarily transmitted by *Culex tritaeniorhynchus* mosquito, with pigs and aquatic birds serving as amplifying hosts [1]. JEV is endemic across much of Asia, the Western Pacific, and northern Australia [2], with an estimated 45,000 human cases annually [3]. Reported mortality is 20–30%, and 30–50% of survivors develop long-term neurological sequelae [4]. For travelers from non-endemic regions, JE is considered low risk (< 1 case per million travelers per month); however, the risk increases significantly during prolonged rural exposure in tropical destinations, reaching up to 1 case per 50,000 travelers per week [4]. Currently, no antiviral therapy exists; treatment is supportive, and prevention relies on vaccination and mosquito bite avoidance [5]. Most JEV infections are asymptomatic or cause only mild viral-like illness. In cases progressing to JE, patients initially develop fever, malaise, and vomiting, followed by acute encephalitis with altered mental status. Severe cases may develop focal neurologic complications, including Parkinsonism, presenting as a resting tremor, mask-like facies, and cogwheel rigidity [4].

## Clinical case

A 70-year-old Lao male with a past medical history of multiple myeloma (MM) on chemotherapy, hypertension, and heart failure with preserved ejection fraction presented to the emergency department (ED) in the middle of June 2023, with an acute onset of generalized weakness and poor appetite. Our patient was diagnosed with multiple myeloma in February 2022 and was recently started on chemotherapy in February 2023, which was later discontinued because of the patient’s prolonged trip to Laos. His oncologist only approved a 6-week trip to Laos, but the patient stayed longer and returned to the California Central Valley at the end of May 2023. His immunization status was unclear.

The patient presented to the same ED a few days prior with a 3-week history of shortness of breath, found to have a hemoglobin of 5 gm/dL, and subsequently transfused with pRBCs and discharged home. Soon after, he presented again to the same ED with shortness of breath and was found to have elevated BNP and pulmonary edema on chest imaging. He was admitted for an acute exacerbation of chronic congestive heart failure, treated with intravenous diuretics, and discharged home. After the second discharge, the patient’s family noticed his increased confusion, loss of appetite, and sudden change in functional status regarding ambulating and using the restroom. According to the family, at baseline, the patient is alert and oriented to person, place, and time, and fully independent.

**Fig. 1:**
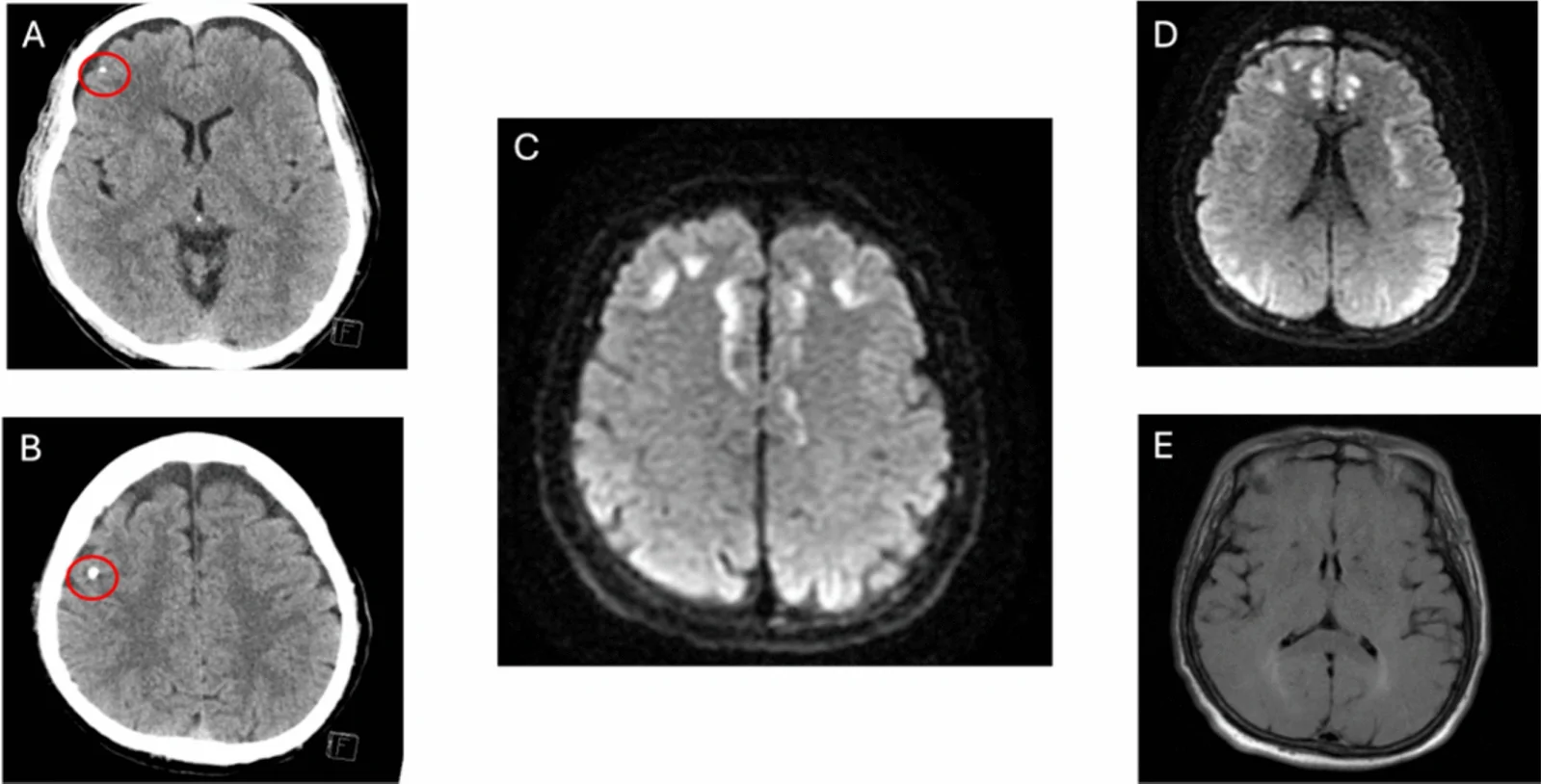
Patient’s brain imaging. **A** and **B** Computed Tomography (CT) images revealing calcifications depicting old neurocysticercosis (indicated by red circles). **C** DWI image showing hyperintense enhancements in the bilateral frontal lobe cortices with punctate microhemorrhages. **D** Diff usion-Weighted Imaging (DWI) image revealing hyperintense left insular enhancement. **E** T2-weighted Fluid-Attenuated Inversion Recovery (T2-FLAIR) image showing no thalamic involvement. CT: Computed Tomography; DWI: Diffusion-Weighted Imaging; T2-FLAIR: T2-weighted Fluid-Attenuated Inversion Recovery

In the ED, the patient was confused and could not provide his own history. His blood pressure was 120/63 mmHg, heart rate 86 beats/minute, respiratory rate 20 breaths/minute, oxygen saturation of 94% on room air, and temperature of 37.3 °C (Table 1). His initial labs were notable for a white blood cell count of 3.9 × 10^6/L, hemoglobin of 9.1 g/dL, platelets of 134,000/uL, creatinine of 1.6 mg/dL, BUN of 18.5 mg/dL, and BNP of 1438 pg/mL. Electrocardiogram (EKG) revealed normal sinus rhythm. Computed Tomography (CT) head without contrast was negative for intracranial hemorrhage or mass effects, but did show calvarial lytic lesions, concerning for metastatic multiple myeloma and cerebral calcifications, concerning for remote neurocysticercosis infection (Fig. 1). Chest imaging showed cardiomegaly with pulmonary vascular congestion and osseous myeloma, and the patient was admitted with an initial diagnosis of acute metabolic encephalopathy secondary to pneumonia and started on intravenous ceftriaxone, azithromycin, and vancomycin.

**Table 1:** Presenting vitals

Vitals	Results
Temperature (°C)	37.3
Heart rate (breaths per minute)	86
Respiratory rate (respirations/minute)	20
Blood pressure (mmHg)	120/63
Oxygen saturation	97
Oxygen method	Room Air
Weight (kg)	65
EKG	Normal sinus rhythm without abnormalities

*EKG* electrocardiogram

While being treated for an infectious etiology, a broad neurologic workup was conducted. Both the neurology and oncology teams were informed of the patient’s admission and participated in his management. A second suspected diagnosis was leptomeningeal involvement of his multiple myeloma, and further studies were sent for evaluation (Tables 2 and 3).

**Table 2:** Laboratory findings

Labs	Results	Reference range
Complete blood cell count		
WBC (white blood cells) × 10^6 /L	3.9	4.0–10.0
Red blood cell count (million/uL)	2.95	4.3–5.9
Hemoglobin (g/dL) (Hgb)	9.1	13–17.0
Hematocrit (% (L))	23.8	39–49
PLT (platelet count) × μ/L	134,000	150–400
International normalized ratio (INR)	1.5	
Partial thromboplastin time (PTT)	60.1	25.1–36.5
Prothrombin time (PT)	17.8	9.4–12.5
General chemistry/metabolic Panel		
Sodium (mmol/L)	141	136–145
Potassium (mmol/L)	4.4	3.5–5.1
Calcium (mg/dL)	9.1	8.4–10.5
Glucose (mg/dL)	99	70–105
Blood urea nitrogen (mg/dL)	18.5	8.4–25.7
Creatinine (mg/dL)	1.6	0.7–1.3
Albumin (g/dL)	1.7	3.5–5.0
Alanine aminotransferase (ALT) (units/L)	29	0–55
Aspartate aminotransferase (AST) (units/L)	47	5–34
Lipase (units/L)	27	8–78
Ammonia level	53	18–72
Lactic acid (mmol/L)	1	0.5–2.0
Thyroid Stimulation Hormone (TSH)	1.9063	0.35–4.94
Vitamin B12 (pg/mL)	717	213–816
Folic acid (ng/mL)	7.8	7.0–31.4
Venous Blood Gas (VBG)		
Partial Pressure of Carbon Dioxide (mmHg)	29	35–48
HCO3 (mmol/L)	23.7	22.0–29.0
Base Excess (mmol/L)	1.2	− 2.0–2.0
Lactate (mmol/L)	1.2	0.5–2.0
Oxyhemoglobin (%)	89	95.0–98.0
Carboxyhemoglobin (%)	3.4	0.5–1.5
Methemoglobin (%)	0.7	0.0–1.5

**Table 3:** Cytology and serology

Tests	Results
Microbiology/serology panel	
COVID-19/SARS-COV-2 PCR	Negative
Influenza A	Negative
Influenza B	Negative
Adenovirus	Negative
Human metapneumovirus	Negative
Parainfluenza Type I	Negative
Parainfluenza Type 2	Negative
Parainfluenza Type 3	Negative
Parainfluenza Type 4	Negative
Corona 229	Negative
Corona OC43	Negative
Corona NL63	Negative
Corona HKU1	Negative
Rhinovirus/enterovirus	Negative
Respiratory syncytial virus	Negative
Bordetella pertussis	Negative
*Chlamydophila pneumoniae*	Negative
*Mycoplasma pneumoniae*	Negative
Urine analysis	Negative
Cerebrospinal Fluid CSF	
CSF glucose	78 mg/dL
CSF protein	349 mg/dL
CSF volume	9 mL
CSF color	Pink
CSF clarity	Hazy
CSF RBC	10,360 cells/UL
CSF WBC	5 cells/UL
CSF Segs	80%
CSF lymph	16%
CSF monocyte	4%
CSF eosinophil	0%
Cerebral spinal fluid microbiology	
Epstein–Barr virus PCR	Negative
Varicella-zoster virus PCR	Negative
Aquaporin-4 (AQP4) NMO-IgG Ab	Negative
Coccidioides antibody	Negative
*Escherichia coli* K1 PCR	Negative
*Haemophilus influenza* PCR	Negative
*Listeria monocytogenes* PCR	Negative
*Neisseria meningitidis* PCR	Negative
*Streptococcus agalactiae* PCR	Negative
*Streptococcus pneumoniae* PCR	Negative
Cytomegalovirus (CMV) PCR	Negative
Enterovirus PCR	Negative
Human herpesvirus 6 (HHV-6) PCR	Negative
Herpes simplex virus 1 (HSV-1) PCR	Negative
Herpes simplex virus 2 (HSV-2) PCR	Negative
Human parechovirus PCR	Negative
Varicella-zoster virus (VZV) PCR	Negative
*Cryptococcus neoformans*/*gattii* PCR	Negative
West Nile Virus IgM	< 0.90

*PCR* polymerase chain reaction, *CSF* cerebrospinal fluid, *RBC* red blood cells, *WBC* white blood cells, *NMO* neuromyelitis optica, *IgG* immunoglobulin g, *IgM* immunoglobulin m

On the second day of admission, our medicine team noted significant waxing and waning of the patient’s mentation. Earlier in the day, the patient was non-verbal and responsive only to painful stimuli; however, on reassessment in the afternoon, the patient made efforts to leave his bed and answered a question verbally. Additionally, he developed fevers with a maximum temperature of 39.4 °C and had slow, rigid movements. The next day, he was obtunded and was transferred to the Intensive Care Unit (ICU) with concerns for protecting his airway. On his fourth hospital day, the patient fell into a comatose state requiring intubation for airway protection. Prior metabolic and infectious workups had returned negative, and an Magnetic Resonance Imaging (MRI) brain with contrast was done, which revealed the presence of an acute infarct in the right frontal lobe without any meningeal abnormality.

Despite the presence of an acute stroke, further investigation was warranted as the size and location of the infarct would not explain the severity of his encephalopathy. On the sixth hospital day, he continued to be in a comatose state. A lumbar puncture was then completed to investigate a broad differential, including endemic infectious conditions in the California Central Valley, rheumatic and immunologic conditions, and malignancy. After reassessing the patient’s clinical decline, risk factors, and potential exposures, a brief literature review through a web browser search revealed reports of similar case presentations attributed to JEV. A decision was made to send a follow-up JEV serum study.

Our patient continued to deteriorate despite antibiotic treatment and supportive efforts. He made minimal neurologic recovery and was subsequently found to have a positive IgG titer for JEV and small IgM. These findings were consistent with recent or active JEV infection. Given his declining state, a decision was made to pursue inpatient hospice care. Our patient passed thereafter.

## Discussion

This case highlights the diagnostic complexity of acute encephalopathy in an immunocompromised traveler. The patient’s prolonged stay in rural Laos, combined with underlying multiple myeloma, significantly increased his susceptibility to severe infection. Immunocompromised individuals are known to be at higher risk for severe outcomes from travel-related infections [4], and impaired immune function likely contributed to his rapid clinical deterioration.

After inoculation, JEV replicates in skin macrophages and dendritic cells before triggering innate immune responses. If uncontrolled, adaptive immunity is necessary with CD4 + T-cell activation, production of neutralizing antibodies, and CD8 + cytotoxic clearance of infected cells. JEV can evade these defenses and enter the central nervous system via the blood–brain barrier through brain capillary endothelial cells or the choroid plexus, leading to infection of neurons, encephalitis, and neuronal apoptosis [6–8]. While both mechanisms have been shown in mouse models, further research is needed to understand the exact mechanisms [7]. We believe both mechanisms could have been at play in our patient. Additionally, the patient’s underlying multiple myeloma and subsequent chemotherapy further impaired immunity through hypogammaglobulinemia, T-cell dysfunction, and immunosuppressive cytokine signaling. This immunologic vulnerability and impaired humoral and cellular immune responses may explain the rapid progression to coma despite supportive care, facilitating rapid disease progression and poor outcome. Hematologic malignancy likely impaired both humoral and cellular immune responses, limiting his ability to control a viral infection. Coinfection with neurocysticercosis may increase the likelihood of worsened neurologic outcomes, as prior studies suggest an association between these conditions and increased disease severity [9]. Pigs are important carriers and amplifiers of JEV because they are susceptible and exhibit mild clinical symptoms, allowing them to transmit the virus in a vector-free manner through mucosal contact and droplets. Therefore, infected pigs may serve as JEV reservoirs, contributing to year-round JEV circulation in temperate climates and causing epidemics during the warmer, wetter seasons when mosquitoes are present. Increased pig farming in endemic regions, with larger animal numbers, may therefore increase the risk of JEV transmission and human infections. By monitoring JEV trends in swine, timely information can be acquired and used to better predict JEV epidemics among humans–promoting effective prevention and control of human JEV infections.

Neuroimaging findings in this case were atypical. While Japanese encephalitis classically involves the thalami, our patient demonstrated frontal and insular cortical involvement without thalamic lesions. This unusual pattern may delay recognition and broaden the differential diagnosis [9, 10].

A limitation of this case report is the testing conducted to confirm the diagnosis. The gold-standard JEV test, plaque reduction neutralization testing (PRNT), could not be performed because our patient’s condition had significantly declined by the time the serum results were available [11]. PRNT offers high sensitivity and specificity; however, it is time-consuming and labor-intensive, and is available only at a few centers, limiting its clinical use as a confirmatory test [11]. While JEV IgM testing is frequently used as an alternative, it can show some cross-reactivity with other closely related flaviviruses. Namely, the Dengue and West Nile Virus. Thus, CSF testing is preferred as serum IgM elicited against other flaviviruses does not cross into the CSF [12]. Another potential diagnosis is a severe dengue infection, which we were unfortunately unable to test for in time. Dengue is rarely associated with encephalopathy or encephalitis and can mimic JE [13]. However, JEV is known to target the Central Nervous System (CNS) directly and is more likely to present with Parkinsonian features, coma, and death, as seen in this patient [13].

## Conclusion

In immunocompromised patients with acute encephalopathy, clinicians must broaden the infectious disease workup to include pathogens relevant to the patient’s home country and recent travel. A limitation of this case is the patient’s complex medical history, which complicates the determination of JE as the primary etiology. Diagnostic certainty is further limited because testing occurred weeks after returning to the United States (US); however, JEV IgM can remain detectable up to 30–90 days after onset [14]. With the lack of answers from extensive testing and a clinical picture consistent with JE, we suspect that JEV contributed to his decline. However, confirmatory testing could not be obtained due to his rapid deterioration. Neuroimaging revealed frontal cortex and left insular involvement without thalamic lesions-mimicking temporal lobe predilection seen in HSV encephalitis [15, 16], though HSV testing was negative (Fig. 1). Such findings have not been reported in JE, which could suggest an atypical presentation. While thalamic lesions on CT/MRI have 23% sensitivity and 100% specificity for JE, these may not appear until 10–20 days, limiting early diagnostic neuroimaging [9, 10]. This case highlights the importance of integrating clinical, laboratory, and imaging findings to inform diagnosis and support families.

## Data Availability

N/A.
